# Extracellular Vesicles and Their Role in the Spatial and Temporal Expansion of Tumor–Immune Interactions

**DOI:** 10.3390/ijms22073374

**Published:** 2021-03-25

**Authors:** Simone Lipinski, Katharina Tiemann

**Affiliations:** 1 University Cancer Center Schleswig-Holstein (UCCSH), University Hospital Schleswig-Holstein, 24015 Kiel, Germany; 2 Institute for Hematopathology, Fangdieckstr. 75, 22547 Hamburg, Germany

**Keywords:** extracellular vesicle, tumor–immune microenvironment, microbiome, physical activity, tumor evolution, innate immune responses, PD-L1, liquid biopsy, diagnostics

## Abstract

Extracellular vesicles (EVs) serve as trafficking vehicles and intercellular communication tools. Their cargo molecules directly reflect characteristics of their parental cell. This includes information on cell identity and specific cellular conditions, ranging from normal to pathological states. In cancer, the content of EVs derived from tumor cells is altered and can induce oncogenic reprogramming of target cells. As a result, tumor-derived EVs compromise antitumor immunity and promote cancer progression and spreading. However, this pro-oncogenic phenotype is constantly being challenged by EVs derived from the local tumor microenvironment and from remote sources. Here, we summarize the role of EVs in the tumor–immune cross-talk that includes, but is not limited to, immune cells in the tumor microenvironment. We discuss the potential of remotely released EVs from the microbiome and during physical activity to shape the tumor–immune cross-talk, directly or indirectly, and confer antitumor activity. We further discuss the role of proinflammatory EVs in the temporal development of the tumor–immune interactions and their potential use for cancer diagnostics.

## 1. Introduction

Extracellular vesicles (EVs) are nanosized membrane-enclosed vesicles that are released by almost all cell types. EVs carry lipids, proteins, metabolites and nucleic acids that are derived from their cell of origin [1]. They interact with recipient cells and have the capacity to induce cellular reprogramming, which renders them as an important intercellular communication tool [2]. In this context, it has emerged that EVs play a decisive role in cancer initiation and progression. EVs derived from tumor cells are linked to several of the hallmarks of cancer [3], including sustained proliferative signaling, resistance of cell death, angiogenesis, invasion and metastasis [4]. Furthermore, they are key drivers of immune escape mechanisms by promoting an immunosuppressive environment, which in turn is licensed by suppressive EV cargo molecules from the parental cancer cell [5]. Apart from tumor cells, EVs are secreted by most immune cell types such as B and T cells, natural killer cells and professional antigen presenting cells such as dendritic cells and macrophages [6]. Immune cells are a critical component of the tumor microenvironment and the modulation of their function plays a vital role for tumor progression or regression. EVs derived from immune cells display immune-regulatory properties identical to their parental cells. This includes the ability to deliver antigens or proinflammatory cytokines to recipient cells. Thus, they are capable of driving inflammatory processes at the tumor site or of mediating immune suppression, which makes EV-mediated communication a highly dynamic and complex process.

The aim of this review is to provide an overview of the EV-mediated mutual regulation between tumor and immune cells in the tumor microenvironment and beyond. We present recent findings that suggest that EVs can transmit antitumorigenic effects from the periphery as effective long-distance mediators, particularly from the microbiome and in response to physical activity. Another focus is on the role of proinflammatory EVs that mediate innate immune responses and their impact on the temporal dynamics of the tumor–immune interactions. Finally, we will discuss perspectives for the potential use of EVs in the diagnostic routine. Of note, we are aware of the fact that EVs can be classified into different subcategories according to their size, composition and origin, and that due to the lack of specific markers for each subtype, nomenclature in the literature has been imprecise. In this review, we will refer to the generic term “EVs” for vesicles including exosomes and small EVs, as suggested by the International Society for Extracellular Vesicles [7].

## 2. EVs Expand the Tumor–Immune Interactions

**Figure 1:**
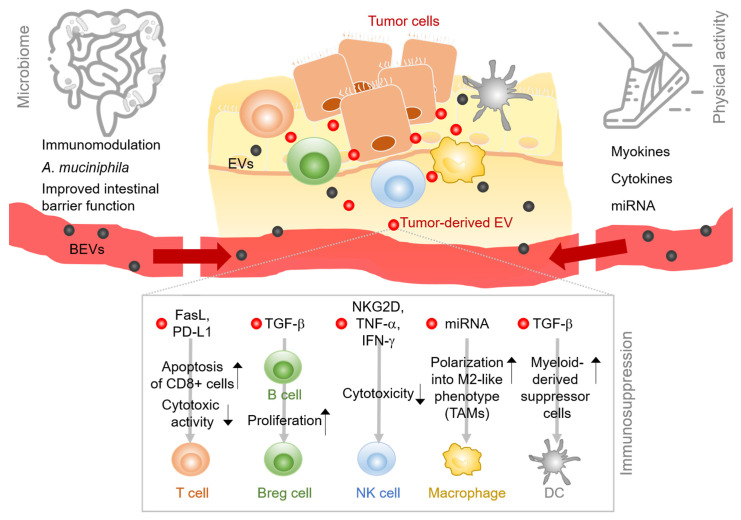
Extracellular vesicles (EVs) expand the tumor–immune interactions. In the local tumor microenvironment, EVs mediate intercellular communication between immune cells and tumor cells. Tumor-derived EVs suppress immune cell functions via several mediators and mechanisms. EVs derived from remote sources have the potential to shape the tumor–immune interactions in the tumor microenvironment directly or indirectly by either bacterial EVs (BEVs), derived from the host microbiome (left side) or by factors released during physical activity (right). Abbreviations: BEVs, bacterial extracellular vesicles; EVs, extracellular vesicles; FasL, fas ligand; PD-L1, programmed death-ligand 1; TGF-β, transforming growth factor beta; Breg cell, regulatory B cell; NKG2D, natural killer group 2D; TNF-α, tumor necrosis factor alpha; IFN-γ, interferon gamma; NK cell, natural killer cell; TAM, tumor-associated macrophage; DC, dendritic cell.

Cancer cells are embedded in a network of nonmalignant cells, vessels, lymphoid organs or lymph nodes and noncellular components like metabolites, signaling molecules and extracellular matrices, which collectively form the tumor microenvironment (TME) [8,9]. The TME and the tumor cells constantly and bidirectionally influence one another and the net outcome of this interaction largely contributes to the determination of the tumor phenotype. Depending on the cellular and molecular composition of the tumor microenvironment, tumor cell growth can be suppressed and the malignancy reversed [10] or corrupted signals from the TME enable and sustain tumor growth and promote its invasion and spreading [3]. However, the conceptual view of the TME as the proximate surrounding of the tumor cells, exclusively accounting for cancer progression or regression, has recently been challenged [11]. Laplane and colleagues suggested to expand the TME-centric view to the “tumor organismal environment (TOE) level in order to encompass components located beyond the TME. These peripheral factors comprise systemic immune components, extracellular vesicles and the microbiota [12,13]. For some of these elements, the distance to the tumor site does not seem to interfere with the bidirectionality of the crosstalk. For instance, gut microbiota composition appears to affect oncogenesis and tumor progression both locally and systemically, for instance via bacterial metabolites [14]. Extracellular vesicles are a prototypical example of complex systemic components. They are neither limited in their composition nor in the distance of their place of origin. In fact, they can interact and communicate with tumor cells anywhere in the body. Vice versa, EV secretion from the tumor cell can act either directly on adjacent or on peripheral immune cells. According to the concept of Laplane and colleagues [12,13] we propose that EVs expand the tumor–immune interaction to a complex systemic process. The concept of the spatially expanded tumor–immune cell crosstalk that is promoted by EVs is depicted in Figure 1.

### 2.1. EVs in the Tumor-Immune Environment Cross-Talk

Tumor cells in the cancer microenvironment are commonly interspersed with immune cells including T cells, B cells, natural killer (NK) cells, macrophages, and dendritic cells (DC). In the histopathological evaluation of the cancer tissue, the presence of innate and adaptive immune cells indicates cancer progression and points towards an immunosuppressive environment [15]. However, in the early stages of tumor development, when innate and adaptive immune responses have not been affected by the tumor, effector immune cells such as natural killer (NK) and CD8+ T cells destroy malignantly transformed cells [16]. During all phases of tumor growth, the mutual crosstalk between tumor and immune cells determines the tumor’s phenotype and ultimately the patient’s outcome [17]. In this dynamic intercellular communication scenario, EVs are now regarded as critical regulators, although the specific mechanisms by which they function are complex and far from being fully understood.

#### 2.1.1. T Cells

T cells are the most common tumor-infiltrating lymphocytes in the TME and are major contributors to antitumor immunity. T cell-derived EVs carry characteristic EV marker proteins such as the membrane tetraspanins CD63 and CD81, cytoskeleton and microtubule-related proteins, but also proteins relevant for T cell immune responses such as FasL and Apo2L/TRAIL or CD95L, components of the TCR/CD3 complex and specific integrins [18]. It was reported that EVs from normal human T cell blasts contained HLA proteins, β2-microglobulin and many CD proteins (CD2, CD5, CD46, CD48, CD58, CD44, CD38, CD6, CD97 and Fas/CD95), whereas these proteins were lost in EVs derived from leukemic cells [18]. In a proteomic study, EVs derived from either resting or activated T cells were analyzed and compared. While proteins related to cytoskeleton organization were enriched in EVs derived from resting T cells, pathways like immune surface receptor signaling, translation and peptide metabolic process were associated with activated T cells [19]. Apart from proteins, the transfer of genomic and mitochondrial DNA [20] and miRNA [21] via T cell EVs has been described. In the context of tumor biology, a role for CD8+ T cell EVs has been demonstrated in the prevention of metastasis. Healthy mice were found to release cytotoxic EVs from activated CD8+ T cells that induced the apoptosis of mesenchymal tumor stromal cells, thereby attenuating tumor invasion and metastasis [22].

It has been demonstrated that EVs derived from tumor cells from various entities mediate immune suppressive effects on T lymphocytes by impairing the activation of effector T cells, either by inducing apoptosis or by diminishing their cytotoxic activity [23]. For instance, when plasma-derived EVs from glioblastoma patients were cocultured with T cells, T cell activity was decreased and lymphocyte migration was hindered [24]. EVs that were isolated from the plasma of melanoma patients inhibited CD69 expression, induced apoptosis and suppressed the proliferation of CD8+ T cells compared to EVs that were isolated from healthy controls [25]. Mechanistically, Chen et al. demonstrated that circulating EVs from melanoma patients and from an in vivo mouse model contribute to tumor immune evasion. They found that melanoma cells released PD-L1-positive EVs, which suppressed CD8+ T cell function via PD-1 and promoted melanoma progression. Treatment with anti-PD-1 immune checkpoint inhibitors reversed the EV-PD-L1-mediated T cell inhibition. Further, the level of PD-L1 on circulating EVs correlated with clinical response to therapy and has the potential to serve as a predictive marker by which responders can be distinguished from nonresponders [26].

#### 2.1.2. B Cells and Plasma Cells

B cells and plasma cells in the tumor microenvironment or in tumor-draining lymph nodes were shown to effectively contribute to the antitumor response. They secrete tumor-specific antibodies and modulate the function of other immune cells [27]. EVs derived from B cells contain histocompatibility complex (MHC) class I and class II and costimulatory proteins, heat shock proteins, integrins and adhesion molecules as well as different types of antigens [28]. The uptake of B cell-derived EVs was shown to be mediated by CD169 on the EV surface, which promoted T cell responses and cytokine production by MHC-II [29,30]. In addition, EVs from B cells were shown to potently induce cytotoxic T lymphocyte responses [31].

In the tumor microenvironment, B cell-derived EVs can exert either pro- or antitumor effects, depending on the environmental conditions and the tumor cell-instructed reprogramming, which can also be mediated by EVs. For example, it was demonstrated that EVs from tumor cells can hijack supportive B cell function in the spleen. EVs from mycoplasma-infected tumor cells induced B cell-dependent IL-10 production, which led to T cell inhibition [32]. EVs that were isolated from surgically removed esophageal cancer cells were shown to induce the differentiation of naïve B cells into TGF-β-producing regulatory B cells (Bregs). Bregs in turn mediated immune suppressor functions on CD8+ T cell proliferation [33]. Melanoma-derived EVs were shown to disseminate via lymph and penetrate lymph nodes, where they activated tumor-enhancing B cell immunity. Under physiologic conditions, however, this circuit was prevented by CD169+ macrophages that interacted with the EVs and limited their spreading, thereby suppressing the tumor-promoting B cell phenotype [34].

#### 2.1.3. NK Cells

NK cell-mediated cytotoxicity is an important antitumor defense mechanism. NK cells were shown to constantly release EVs that exert antitumor activity on several cancer-derived cell lines including T cell leukemia, Burkitt lymphoma, metastatic breast cancer, adenocarcinoma and neuroblastoma [35,36,37]. EVs from NK cells comprised the characteristic EV markers CD63, tsg101, CD81 and CD9 and the parental NK cell marker NKG2D. They further contained the cytotoxic effectors perforin, granulysin, granzymes A and B as well as nucleic acids [36,38]. NK EV-mediated killing mechanisms of recipient cells activate several signaling cascades including caspase-dependent and independent cell death pathways and ER stress [39]. Importantly, it was demonstrated that NK cell EVs still maintain their stimulatory and cytotoxic function under immunosuppressive conditions, for instance in the presence of TGF-β, which may represent a strategy to overcome immune escape mechanisms in the tumor microenvironment [37,38]. Still, tumor-educated EVs can compromise NK cell function and limit their capacity to directly kill tumor cells. EVs from primary pancreatic cancer cells or highly metastatic pancreatic cancer cell lines that were cocultured with NK cells lowered the cytotoxic potential and downregulated NKG2D, CD107a, TNF-α and INF-γ in NK cells [40]. Similar effects were reported in acute myeloid leukemia, T and B cell leukemia/lymphoma, mesothelioma and serval other cancer cell lines [41,42,43]. The NKG2D ligand was found to be released from tumor EVs and downregulate NKG2D expression, resulting in attenuated activity of the NK cells [41,42,43].

#### 2.1.4. Professional APCs

Professional APCs, such as dendritic cells (DC) and macrophages, release EVs to induce immune responses in recipient cells, which directly or indirectly affect tumor cells. The first description of an EV-transmitted immune response to intracellular pathogens was shown in macrophages. EVs that were isolated after macrophage infection (mycobacteria, salmonella or toxoplasma) and then transferred to uninfected macrophages stimulated a proinflammatory response. The intranasal application of the proinflammatory EVs into mice led to neutrophil and macrophages’ recruitment in the lung, and the presence of LPS within the EVs was attributed to the observed effects [44]. It is well known that macrophages can promote cancer progression. The subgroup of M2d macrophages, also referred to as tumor-associated macrophages (TAMs), release IL-10 and vascular endothelial growth factor (VEGF), which stimulates angiogenesis and tumor growth [45]. In analogy, TAM-derived EVs (TAM-EVs) have been shown to mediate protumorigenic effects. A study by Zhou at al. found that TAM-EVs had a suppressive effect on CD4+ T cells which was mediated by EV miRNAs miR-21-5p and miR-29a-3p. The miRNAs inhibited the STAT3 signaling pathway and induced and imbalance in Treg/Th17 cells, generating an immunosuppressive microenvironment that facilitated epithelial ovarian cancer progression and metastasis [46]. Another TAM-EV-derived miRNA, miR-501, targeted the tumor suppressor gene TGFBR3 and led to the activation the TGF-β pathway, facilitating the development of pancreatic ductal adenocarcinoma [47]. These results are in contrast to a study by Cianciaruso and colleagues. They performed proteomic and lipidomic profiling of TAM-derived EVs and explored their effects on cancer cells and T cells. According to their analysis, TAM-EVs had molecular profiles associated with a Th1/M1 polarization signature, enhanced inflammation and immune response, and might stimulate antitumor immunity [48]. Of note, the divergent results could partly be attributed to differences in EV isolation techniques since ultracentrifugation followed by polymer-based precipitation was used by Yin and colleagues, whereas sequential ultracentrifugation was applied by Cianciaruso and colleagues.

Accumulating evidence suggests that EVs derived from tumor cells immunomodulate macrophages and support their tumor-friendly effects. For instance, TP53-mutated colon cancer cells selectively released EVs enriched in miR-1246. When macrophages fused with these tumor EVs, miR-1246 triggered cellular reprogramming. As a consequence, the reprogrammed macrophages showed increased levels of oncogenic factors such as TGF-β activity and IL-10 release and were associated with poor survival rates [49]. Of note, another study reported that uptake of the oncogenic miR-1246 from ovarian cancer EVs selectively occurred in M2-type macrophages, but not in M0-type naïve macrophages, indicating that protumorigenic priming in the tumor microenvironment plays a critical role [50]. Hypoxia in the tumor microenvironment is associated with antitumor immunity and enhanced tumor aggressiveness [51]. Along these lines, a recent study showed that EVs, retrieved from hypoxic ovarian cancer cell lines delivered miRNAs which resulted in M2 macrophage polarization and the promotion of tumor proliferation and migration [52]. Further evidence for tumor EV-derived miRNAs and their potential to shift macrophage phenotypes towards immunosuppression, was shown in EVs from glioblastoma and liposarcoma [53,54].

Taken together, the role of EVs in orchestrating immune responses within the tumor microenvironment and in the periphery is becoming increasingly evident. However, the resolution of their specific contributions and involved mechanisms is highly complex. Multiomics technologies, which are currently being used to tackle molecular mechanisms at the single cell level or in host–microbe interactions, need to be applied to the level of EVs. These approaches will unravel the contribution of the EV cargo in different immune contexts and in different stages of tumor evolution. Ultimately, the knowledge must be translated into novel diagnostics and therapeutic approaches.

### 2.2. EVs in the Microbiota—Gut Barrier Tumor Cross-Talk

The human body is colonized by a diverse microbial community, collectively referred to as the microbiome. The highest microbial density is found in the gut, especially in the large intestine, where approximately 500–1000 different bacterial species make up 10^11^ to 10^12^ bacterial cells/gram colon content [55]. Like eukaryotic cells, Gram-positive and Gram-negative bacteria release a heterogenous mixture of membrane vesicles, so-called bacterial extracellular vesicles (BEVs) [56,57]. Their cargo includes membrane-bound and periplasmic proteins, enzymes and toxins, polysaccharides, metabolites, DNA and RNA and peptidoglycans [58]. As the latter suggests, they characteristically contain conserved microbe-associated molecular pattern molecules (MAMPs) derived from the bacterial cell membrane. These include lipopolysaccharide (LPS) in Gram-negative BEVs and lipoteichoic acid (LTA) in Gram-positive BEVs [59]. In the human body, the presence of MAMPs is detected by pathogen recognition receptors (PRRs) expressed by host cells, particularly immune cells. LPS and LTA activate an immune response via the Toll-like receptors (TLRs) TLR4 and TLR2, respectively. Evidence that BEVs from the intestinal microbiome cross the intestinal epithelial barrier and enter the circulation was recently provided [58,60]. In a mouse study, the uptake and distribution of orally administered BEVs from the commensal gut bacteria *Bacteroides thetaiotaomicron* was assessed under physiologic conditions. The transmigration of BEVs into the circulation and lymphatic systems was reported to occur for a small portion of BEVs. They were detected in heart, lung and liver tissue [61]. The potential of BEVs to enter the systemic circulation correlates with gut permeability. Since intestinal permeability is affected by the intestinal microbial composition, the potential of BEVs to transmigrate into the circulation is equally dependent on it. In cancer, the structural composition of the gut microbiome changes and most likely affects the amount and molecular makeup of transmigrating BEVs. The dysbiotic shifts in cancer have been shown to contribute to oncogenesis and cancer progression [62]. Additionally, the individual structure of the gut microbiota was found to influence antitumor immunosurveillance and to determine the response to immunotherapy in cancer patients [63]. The mechanistic insights are still limited; however, it is assumed that both direct and indirect effects play a role, highlighting the dynamic interactions between microbes, lifestyle factors (diet, drugs) and the host [64,65]. In analogy to this, the influence of BEVs on the tumor cell, growth, microenvironment and immune response is unknown and expected to be highly complex. Additionally, although mechanistic studies of how BEVs influence oncogenesis and tumor progression are still missing, current knowledge suggests that BEVs interact with immune cells to regulate inflammatory responses [56]. These data can serve as a reasonable proxy for their putative role in cancer.

Most recently, the group of An Hendrix provided evidence that LPS-positive BEVs circulated in the plasma of patients with clinically well-defined intestinal barrier dysfunction. Isolated BEVs from the plasma of cancer patients who suffered from therapy-induced intestinal mucositis showed significantly increased levels of LPS activity compared to cancer patients without apparent gastrointestinal side effects. Indeed, the level of BEV-associated LPS was significantly correlated with the plasma zonulin levels, which implies disturbed intestinal barrier integrity. Moreover, BEVs activated a TLR4-mediated immune response and induced the secretion of proinflammatory cytokines like IL-6, IL-8, MCP-1 and MIP-1α by peripheral blood mononuclear cells [60].

The immunomodulatory effects mediated by BEVs have been shown to depend on the specific characteristics of their parental bacterial cell. BEVs from the gut commensal *Bacteroides fragilis,* containing the immunomodulatory agent PSA, were shown to be sensed by dendritic cells via TLR2. BEVs were internalized by the dendritic cells and induced an immune response leading to the production of regulatory T cells and anti-inflammatory cytokines [66]. BEVs from the opportunistic pathogen *Salmonella typhimurium*, on the contrary, mediated strong proinflammatory effects in professional APCs in vitro. Upon BEV stimulation, macrophages and dendritic cells showed enhanced surface expression of MHC-II and CD86 and increased release of NO, TNF-α and IL-12. Mice that were injected with BEVs developed protective T cell and B cell responses, conferring immunity to infection [67].

Apart from exhibiting immune-modulating properties on host cells, BEVs affect inflammatory diseases [68]. The presence of the symbiotic bacteria *Akkermansia muciniphila* in the intestinal microbiome has been associated with anti-inflammatory effects and benefits for host health [69]. In a mouse model of intestinal inflammation, BEVs derived from *Akkermansia muciniphila* reduced colonic inflammation by lowering levels of inflammatory cytokines and strengthening intestinal barrier function [70,71]. In cultured intestinal epithelial cells, the administration of *A. muciniphila*-derived BEVs resulted in the upregulation of tight junction proteins [72]. Two further studies reported extraintestinal effects of in vivo administered BEVs from *A. muciniphila*. One study found that high-fat diet-induced obesity was ameliorated and that lipid metabolism and inflammatory gene expression were affected by *A. muciniphila*-derived BEVs in mice [73]. Another study reported that BEVs from *A. muciniphila* mediated effects on the serotonin signaling pathways in the colon and brain of mice [74].

Interestingly, *A. muciniphila* links bacterial gut composition to cancer immunotherapy. Routy and colleagues provided evidence for an association between the intestinal colonization with *A. muciniphila* and the efficacy of immunotherapy in cancer patients. They showed that the clinical response to immune checkpoint inhibitors correlated with the relative abundance of *A. muciniphila.* Using a mouse model, they confirmed the beneficial effects of *A. muciniphila.* Initially, germ-free mice were colonized with stool from nonresponder patients, which rendered them less responsive to PD-1 blockade. When the mice were then orally supplemented with *A. muciniphila*, the efficacy of PD-1 blockage could be restored. This effect was shown to be mediated by IL-12, leading to an increased recruitment of CCR9+CXCR3+CD4+ T lymphocytes into mouse tumor beds [75].

Future studies are needed to clarify to which extent single bacterial species, BEVs and/ or bacterial products communicate with cancer cells and cells in the tumor microenvironment. The stability and capacity of BEVs to act remotely from the intestinal tract via direct or indirect mechanisms seems to be a tempting opportunity to engage them as “oncomicrobiotics”. However, when considering BEVs as potential agents to promote beneficial immune responses through optimizing the gut microbiome, it must be taken into account that the intestinal microbiome is constantly shaped by a number of lifestyle factors that will ultimately impact BEV-mediated crosstalk, such as dietary habits, smoking, drugs (especially antibiotic use), sleep, depression and the level of physical exercise.

### 2.3. EVs in Physical Exercise-Tumor Cross-Talk

Physical activity is associated with improved health and reduced risk of many cancer types including breast, esophagus, ovarian and colon cancer [76]. Moreover, an association between the level of physical activity and progression-free survival of patients with metastatic colorectal cancer has recently been described [77]. The protective effects are governed by multiple local and systemic mechanisms, mediated by hormones, metabolites, inflammatory factors, myokines and miRNAs [78]. In the past five years, evidence has accumulated that physical activity and exercise induce the release of EVs that contain functional molecules which are involved in the communication between tissues and organs [79,80,81]. Next to muscle cells, platelets, endothelial cells and leukocytes contribute to the multiple sources of exercise-induced EVs in the blood stream [82,83,84,85]. Accordingly, the circulating mixture of exercise-induced EVs carries a diverse set of cargo proteins, metabolites and miRNAs involved in processes such as angiogenesis, immune signaling and glycolysis, reviewed in [86]. A study in human exercising volunteers confirmed the distinct biological processes [80]. Whitman and colleagues performed a temporal analysis of the plasma EV proteome in response to exercise. They identified over 300 proteins in the plasma EV proteome to be significantly different between exercise and rest. The analysis of significantly enriched GO Terms of biological processes revealed an enrichment for processes ranging from signal transduction to immune cell proliferation [80]. Since it is currently unclear whether there is a link between exercise-induced EVs, improved immune function and anticancer mechanisms, further studies are needed to address the question of whether exercise-induced EVs have the potential to counteract tumor-mediated immunosuppression in the tumor microenvironment. Indeed, Whitman and colleagues postulate that the efflux of EVs into the circulation is a mechanism by which the skeletal muscle releases myokines (cytokines and other peptides that are released by the muscle) independent of the classical secretory pathway [80]. A myokine candidate that has been linked to reduced cancer risk and recurrence is the myokine IL-6 [87]. Pedersen and colleagues found that exercise led to an epinephrine-induced mobilization and redistribution of IL-6-sensitive NK cells to the tumor site. The blockage of IL-6 counteracted intratumoral NK cell infiltration and tumor suppression [87]. Further, the myokines oncostatin M (OSM) and Irisin were shown to decrease breast cancer cell migration and viability in vitro [88,89]. In colon cancer, the myokine SPARC was upregulated in exercising mice and inhibited proliferation and increased apoptosis in colon cancer cells in vitro [90].

Apart from proteins, other factors like miRNA or metabolites, incorporated in EVs, are likely to play a role. It has been reported that several circulating miRNAs may mediate the beneficial effects of physical activity [91,92,93,94]. Amongst them, miR-206 was found to reduce breast cancer cell growth by slowing G1/S transition and inducing apoptosis in vitro. Myoblasts that were stimulated by gravity to simulate physical activity were shown to release miRNA-microvesicles containing miR-206 [91]. Others have found miR-206 to be associated with the modulation of inflammation [95] and the promotion of antiangiogenic effects in exercising breast cancer patients [96]. Evidence that miR-206 localizes to circulating exercise-induced EVs has been presented by Guescini et al. [82]. Contrary to the effects of physical activity, adipose-tissue-derived EVs stimulated melanoma progression in obese human and mice and promoted melanoma aggressiveness via EV-delivered fatty acids [97,98].

Together, these results point to an important mechanism by which exercise-induced EVs alter tumor initiation and progression. More studies are needed to identify the bioactive cargo and decipher the complex mechanisms underlying their beneficial effects.

## 3. EVs in Tumor Development—Role of Immune Responses and Implications for Diagnostics

With the advent of next generation sequencing (NGS) technologies and the fine resolution of mutational dynamics and tumor heterogeneity at the single cell level, the understanding of tumor evolution has been refined. Over the entire course of cancer progression, beginning with tumor initiation and propagation, followed by metastasis and drug resistance to chemotherapy [99], emphasis is now put on defining and targeting the molecular phenotype, such as the mutational status or biomarkers like surface receptors [100]. Although the temporal dynamics of cancer evolution are still not fully understood [101], it is highly likely that EVs play a critical role in the temporal course of cancer development. In this part we focus on the current knowledge of EV-mediated tumor–immune interactions in the temporal development of cancer, particularly in the phases of (i) cancer initiation and propagation and (ii) treatment-induced alterations and resistance. From this, we will discuss the implications for EV-based diagnostic approaches.

### 3.1. EVs in the Promotion of Cell Proliferation and Immune Escape

According to the current understanding, the initiation and progression of cancer is promoted by mutations and genetic alterations that confer a selective advantage to the cell and lead to phenotypic changes, such as increased cellular proliferation and inhibition of apoptosis [102]. These oncogenic driver mutations can result from genomic instability or from DNA damage induced by carcinogens such as the exposure to radiation, smoking or viruses. In addition, chronic inflammation is a procancerous factor that aids in the establishment of a tumorigenic microenvironment [103]. For example, the risk of colorectal cancer is increased in inflammatory bowel disease. *Helicobacter*-induced gastritis, chronic gastritis and Epstein–Barr virus infections are associated with gastric cancer [104]. Recently, Liu and colleagues pointed out that EVs derived from gastric cancer promoted an immunosuppressive microenvironment by specifically increasing suppressive cytokine secretion which impaired immune function. Consequently, CD8+ T cells and NK cells were decreased and immunosuppressive myeloid-derived suppressor cells (MDSCs) recruited, which facilitated the escape of tumor cells from the host immune system [105].

Inflammation and carcinogenesis share common cellular processes including apoptosis, proliferation and angiogenesis [3]. On the molecular level, these signaling pathways are mediated by transcription factors such as nuclear factor-kappa B (NF-κB) [106] or signal transducer activator of transcription 3 (Stat3) [107] and fueled by cytokines, growth factors and chemokines such as TNF-α, IL-6, IL-8, TGF-β and IL-10. Likewise, reactive oxygen species (ROS) and reactive nitrogen species (RNS) contribute to inflammation-induced carcinogenesis [108]. The concentrations of these mediators are elevated in the tumor microenvironment and extracellular vesicles are significantly involved in their biodistribution. Over a decade ago, Soderberg and colleagues reported that EVs from melanoma cells transferred TNF-α to recipient T cells, which resulted in elevated ROS levels and the disruption of the activation of CD4+ and CD8+ T cells [109]. In multiple myeloma, aberrant plasma cell growth is supported by bone marrow mesenchymal stromal cells (BM-MSCs). It was demonstrated that EVs from BM-MSCs are transferred to multiple myeloma cells. In addition, the level of cytokines, namely IL-6 and MCP-1, as well as further oncogenic proteins and adhesion molecules was significantly elevated in EVs derived from BM-MSCs from diseased patients compared to BM-MSCs from healthy donors. In vitro, the BM-MSC-derived EVs showed divergent roles on tumor growth. While BM-MSC-derived EVs from multiple myeloma patients promoted tumor growth, normal BM-MSC EVs inhibited the growth of multiple myeloma cells [110]. The NF-κB target gene IL-8 was found to be selectively enriched in circulating EVs from the plasma of glioblastoma (GBM) patients and GBM-tumor-bearing mice compared to EVs from unaffected controls. IL-8 was associated with hypoxic regions of GBM xenografts and enriched in in vitro isolated EVs from hypoxic GBM cells compared to EVs from normoxic GBM cells. Since hypoxia correlates with tumor aggressiveness, the authors suggested that circulating EVs in GBM patients represent a potentially druggable target during tumor development [111]. Of note, hypoxia was also described as a factor that led to increased levels of TGF-β and IL-10 in EVs derived from lung cancer cell lines [112].

EV-derived TGF-β has been connected to tumor progression by several studies and EV-TGF-β-mediated signaling plays a well-established role in the induction of the immunosuppressive environment [113]. For instance, the levels of EV-TGF-β were significantly increased in pancreatic cancer patients and coculturing of EVs derived from primary pancreatic cancer cells or cell lines resulted in attenuated NK cell cytotoxicity [40]. Likewise, the suppression of T cell proliferation was mediated by EV-derived TGF-β, interleukin-10 and prostaglandin E2 from hypoxic breast cancer cells [114]. In gastric cancer, macrophages were found to transfer cancer-derived TGF-β and Wnt3 via EVs to surrounding stromal cells, which contributed to the establishment of a protumor microenvironment [115].

Several proinflammatory cytokines such as TNF-α, IFN-α/β, TGF-β and IL-4/6/17/27 have been shown to induce the expression of programed death ligand 1 (PD-L1) in tumor cells and tumor-associated stromal cells [116]. In addition, PD-L1 was found to be regulated by the IFN-γ-JAK1/JAK2-STAT1/STAT2/STAT3-IRF1 axis [117]. The upregulation of PD-L1 on the surface of tumor cells and tumor infiltrating immune cells (TIICs) is known to mediate T cell suppression via programmed cell death protein 1 (PD-1) [118,119]. While the interaction between PD-L1 and PD-1 regulates immune responses and maintains self-tolerance under physiologic conditions, the aberrantly high expression of PD-L1 enables cancer cells of several tumor types to evade the immune surveillance [120]. The blocking of the PD-1/PD-L1 immune checkpoint using monoclonal antibodies has been established as a successful therapy to treat cancer patients [121]. Of note, the clinical response to therapy was found to be independent of the PD-L1 expression on tumor cells, since some patients that were negatively stained for PD-L1 on the tumor surface benefited from PD-1/PD-L1 inhibitor treatment [122,123]. Recent studies have shown that tumor cells release PD-L1 in EVs and that the level of EV-PD-L1 correlates with the level of PD-L1 in the parental tumor cell [26,124,125,126]. Despite the fact that the mechanism of how EV PD-L1 is transferred to recipient cells remains to be elucidated, it has been demonstrated that EVs transport PD-L1 from PD-L1-positive to PD-L1-negative breast cancer cells. In addition, the authors showed that EV-derived PD-L1 bound to PD-1 on T cells and inhibited T cell activation. The transfer of EV-PD-L1 was not limited to T cells and was also shown to occur to macrophages and DCs in vitro [127].

While the mechanisms discussed above play an important role in the promotion of tumor progression, it must be noted that the host is simultaneously employing anti-inflammatory and antitumorigenic strategies. For instance, in the early stages of carcinogenesis, M1 macrophages display antitumor activity [128] and the fine-tuning of NF-κB signaling was shown to balance the antitumor and protumor properties of macrophages [129,130]. In fact, in the early stages of tumorigenesis there is a homeostatic interaction between inflammation, immunity and tumorigenesis. Once cancer cells overcome these balances, they induce spreading and metastasis by actively reeducating their environment [131].

### 3.2. EVs in Intervention-Induced Innate Immune Responses

Tumor cells gain time to grow and progress by turning off immune cell attacks via the mechanisms discusses above. It has been shown that the successful reactivation antitumor immunosurveillance by immunotherapies can be explained by the level of T lymphocytes and inflammatory myeloid cells in the tumor microenvironment [132]. The response to immune checkpoint inhibitors has been linked to a highly infiltrated and inflamed (referred to as “hot”) tumor microenvironment, which displays type I IFN activation, presence of chemokines, cytotoxic effector molecules and CD8+ T cells. In contrast, “cold” tumors, which lack lymphocyte infiltration, are refractory to immunotherapy [133,134]. Current therapeutic approaches therefore try to turn “cold” tumors “hot”. Strategies to sensitize the tumor microenvironment primarily aim to enhance IFN signaling by innate immune cytosolic sensor activation triggered by chemo-, radio- or oncolytic virus therapy [135]. Mechanistically, the cytosolic presence of tumor-derived double-stranded DNA (dsDNA) activates the innate immune cGAS/STING pathway. Upon binding DNA, the cyclic GMP-AMP synthase (cGAS) forms cyclic GMP-AMP (cGAMP). cGAMP then binds to stimulator of interferon genes (STING). This finally leads to the transcription of IFN-β and the induction of several interferon-stimulated genes (ISGs) [136]. Increasing evidence suggests that EVs participate in these processes as active mediators. Recently, Kitai and colleagues demonstrated that breast cancer cells released DNA-containing EVs in response to treatment with the topoisomerase inhibitor topotecan. Using genetically engineered mice, they showed that the EV-DNA exhibited immunostimulatory activity via the cGAS/STING pathway which triggered an antitumor immune response by inducing type I IFN production in DCs [137]. In addition, radiotherapy, which is known to boost cytosolic dsDNA levels in cancer cells, was linked to EV shuttling. Upon irradiation of the breast cancer cells, the EVs were released and transferred dsDNA from the parental tumor cells to DCs. In DCs, this led to STING-dependent IFN-I production. When the irradiation-derived tumor cell EVs were tested in their potential to induce antitumor immune responses, it was found that they mediate tumor-specific CD8+ T cells responses. In mice, irradiation-derived EV conferred protection from tumor progression compared to EVs from untreated cancer cells [138]. However, it has to be noted that, contrarily, EVs from irradiated breast cancer cells fostered a tumor-permissive microenvironment by inducing reactive oxygen species (ROS) and autophagy in primary mammary epithelial cells [139].

Apart from the cGAS/STING pathway, cytosolic dsDNA also activates the AIM2 inflammasome, a multiprotein complex that ultimately leads to the activation of caspase-1, which in turn mediates IL-1β maturation [140]. Lian and colleagues demonstrated that the chemotherapeutic agent irinotecan induced the massive release of dsDNA from the intestine in vivo and in vitro. The dsDNA entered the cytosol of innate immune cells via EVs and induced an AIM2-dependent inflammasome activation and subsequent release of IL-1β and IL-18. They found that IL-1β and IL-18 triggered intestinal inflammation and that either the blockage of EV trafficking or the pharmacological inhibition of AIM2 reversed the chemotherapy-induced intestinal toxicity [141]. Of note, EV-derived IL-1β can induce NF-κB responses in target cells [142] and inhibition of NF-κB signaling has been demonstrated as potential treatment strategy for some entities [143,144]. Along these lines, Kaplanov and colleagues noticed that the neutralization of IL-1β was associated with tumor regression. Using IL-1β -deficient mice they found that antitumor immunity was promoted by IL-12 secretion, which led to CD11b+ DC infiltration in the tumor. When they combined anti-IL-1β treatment with anti-PD-1 checkpoint inhibitors, they observed abrogated tumor progression [145]. Further mechanistic evidence that links inflammasome activation and resistance to anti-PD-1 immunotherapy was provided recently. The immunotherapeutic blockade of PD-1 activated the NLRP3 inflammasome, which in turn led to the recruitment of granulocytic myeloid-derived suppressor cells into the tumor bed. Upon the inhibition of NLRP3 inflammasome activation the tumor infiltration of the suppressive immune cells was reversed and the efficacy of anti-PD-1 immunotherapy was augmented [146]. Together, these studies demonstrate the translational potential for targeting EV-mediated innate immune responses in cancer therapeutic interventions.

### 3.3. Implications for Diagnostics

**Figure 2:**
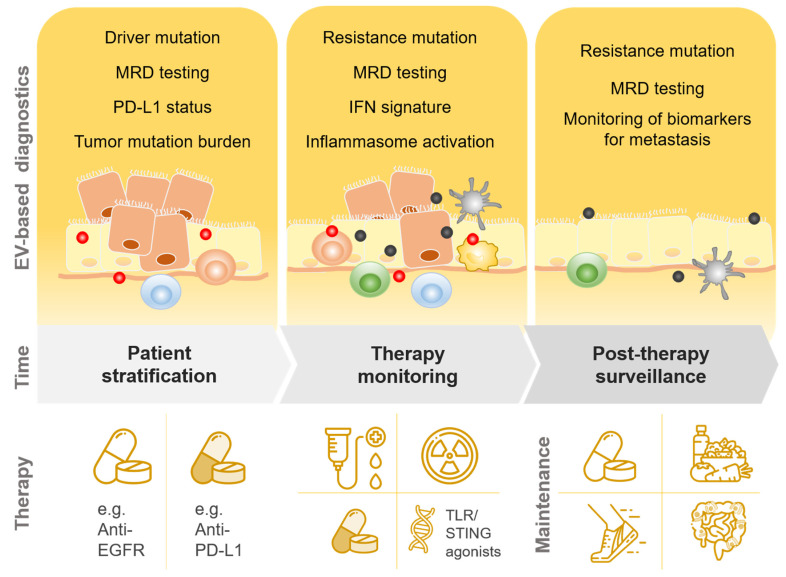
Potential integration of EV-based diagnostics in individualized cancer therapy. Abbreviations: EV, extracellular vesicles; MRD, Minimal residual disease; PD-L1 programmed death-ligand 1; IFN, interferon; EGFR, epidermal growth factor receptor; TLR, Toll-like receptors; STING, Stimulator of interferon genes.

The integration of EV-based diagnostics into clinical routine offers unique potential for noninvasive patient treatment in the age of personalized medicine (Figure 2). Today, cell-free circulating tumor DNA (ctDNA) from biological fluids is already used as “liquid biopsy” to monitor and predict tumor progression and treatment response [147]. In the routine diagnostic laboratory, liquid biopsy ctDNA-based analyses include assessment of tumor-specific mutations, drug resistance, PD-L1 expression and tumor mutational burden [148,149]. However, compared to ctDNA, EVs are more stable in body fluids and blood plasma and extend the targetable classes of biomolecules to proteins, metabolites and RNA [150].

In addition, genomic DNA has been reported to be present in EVs. However, caution is advised here as whether the DNA associated with EVs is more likely to be travelling on the EVs and thus potentially represents an artefact is the subject of current debate. For circulating EVs in the serum of pancreatic ductal adenocarcinoma patients it was shown that isolated EVs contained genomic DNA bearing mutations in KRAS and p53 [151]. Another group demonstrated that dsDNA was present in EVs and represented the whole genomic DNA [152]. Recently, Klump and colleagues compared the diagnostic value of ctDNA to DNA enclosed in EVs. They tested for the BRAF V600E mutation in melanoma patients and cKIT D816V variant in patients with mastocytosis. While their results indicated that total DNA content was elevated in EVs compared to ctDNA, a ten-fold increase in copy numbers was detected in the ctDNA fraction, indicating the importance of ctDNA for diagnostics [153]. A combined assessment of EV-derived nucleic acids and ctDNA has been shown to increase the sensitivity for EGFR mutation detection in plasma from non-small-cell lung carcinoma (NSCLC) patients. In particular, a subgroup of patients with intrathoracic disease (M0/M1a), who show limited levels of ctDNA copies, experienced the largest added value [154].

EVs are involved throughout tumor initiation and progression, and their analysis as circulating biomarkers is therefore subject to the specific spatial and temporal conditions. As suggested by Klump and colleagues, the implementation of liquid biopsies and EV-based diagnostics can be utilized for patients in the form of (i) pretherapeutic screening and patient stratification (ii) monitoring during therapy and (iii) post-therapeutic screening and surveillance [153]. In this context, EV-based liquid biopsy testing could be of potential value when assessing the optimal time point for the induction of immunotherapy. For example, immune checkpoint inhibitors have low response rates and sensitizing strategies help to transform refractory “cold” tumors into responsive “hot” tumors [133,134]. Hence, the monitoring of DNA-containing EVs, which induce an immune response via cGAS/STING and subsequently activate antitumor immune responses [137], could be used to determine the optimal time point for immunotherapy. Likewise, circulating inflammasome components or IL-1β, incorporated in EVs, could provide information about whether a blockade of IL-1β is necessary to improve patient’s health and treatment outcome. In addition, the direct determination of PD-L1 can be useful. For instance, levels of circulating EV PD-L1 were associated with advanced head and neck cancer, metastatic melanoma and poor prognosis in pancreatic cancer, which indicates their usefulness as biomarkers for tumor progression [155,156]. Huang and colleagues reported that they developed a sensitive quantification method for EV PD-L1 that distinguished cancer patients from healthy individuals and correlated with metastasized adenocarcinoma [157]. Approaches like these are urgently needed as several technical challenges currently limit the use of EVs in the diagnostic routine setting, which require rapid and reproducible protocols. In particular, reproducibility and proper EV cleaning methods are currently an issue and the conflicting results in the literature may partially be explained by different EV isolation techniques. At present, the protocols for EV isolation largely differ not only between methods (e.g., ultracentrifugation, density gradient centrifugation, size exclusion chromatography or polymer-based precipitation) but also within a method. For instance, no unified protocol for the specific conditions of differential centrifugation exists. Ultracentrifugation is the most commonly used technique but can lead to impure isolates and thus misleading results due to contaminating molecules in the ultracentrifuged pellet. Other methods might not be applicable to the clinical diagnostic routine setting, for instance the isolation of high-pure EVs by a combination of iodixanol density gradient and ultracentrifugation, which is laborious and time consuming, or commercially available kits using polymer-based precipitation, which are currently intended for research use only. Thus, the standardization, rigor and reproducibility of EV isolation and measurements are a prerequisite for laboratory diagnostics. Once established, they will contribute to unfolding the enormous translational potential of EVs as circulating biomarkers for individualized treatment and diagnostic recommendations.
